# Interkingdom communication 2.0: pheromone-based control between *Bacillus subtilis* and *Saccharomyces cerevisiae*

**DOI:** 10.1007/s00253-026-14069-5

**Published:** 2026-10-15

**Authors:** Julia Döring, Tomislav Vološen, Kai Ostermann, Diana Wolf

**Affiliations:** 1 Faculty of Biology, Group “Biological Sensor-Actuator Systems”, Technical University of Dresden, Dresden, Germany; 2 Faculty of Biology, General Microbiology, Technical University of Dresden, Dresden, Germany; 3 Department of Aquatic Microbiology, Institute of Microbiology, University of Greifswald, Greifswald, Germany

**Keywords:** Pheromone signaling, *Bacillus subtilis*, *Saccharomyces cerevisiae*, Epifluorescence microscopy, Co-culture

## Abstract

**Abstract:**

Microbial communities found in nature are complex and thrive in harmony, consisting of organisms derived from several kingdoms. Hence, they need to be able to communicate by quorum-sensing molecules to co-exist in close proximity. Being able to engineer artificial interkingdom communication for biotechnological approaches, such as degradation of complex biomaterials in one fermenter using controlled signaling between microorganisms, would be beneficial. Therefore, we wanted to establish a proof of principle by enabling the Gram-positive bacterium *Bacillus subtilis* to communicate in a controlled manner with the eukaryotic yeast *Saccharomyces cerevisiae* by exploiting the yeast’s pheromone system. We genetically modified *B. subtilis* to heterologously produce the α-pheromone after bacitracin induction, which in turn would activate the production of the green fluorescent protein mNeongreen upon pheromone recognition by *S. cerevisiae*. Co-cultivation in modified growth medium and induction of pheromone expression was successful and repeatable. Yeast activation and production of mNeongreen was visible not only by analysis but also by epifluorescence microscopy of the co-culture. This study successfully demonstrated controlled interkingdom signaling between microorganisms by leveraging their native communication systems.

**Key points:**

• *Heterologous production and secretion of small peptides in Bacillus subtilis.*

• *Interkingdom communication between bacteria and yeast.*

• *Signaling by artificially produced pheromones.*

**Supplementary Information:**

The online version contains supplementary material available at https://doi.org/10.1007/s00253-026-14069-5.

## Introduction

Communication is crucial in many ways, either for higher eukaryotes or for microorganisms. One important “biotechnological” system relying upon a perfect balance of interaction of different microorganisms is the human gut, supplying its host with nutrients. In the Human Microbiome Project, about 3000 different bacterial species have been identified in human feces (The human microbiome project consortium 2012). An imbalance in this highly complex system might lead to different severe diseases (Manos 2022). To stabilize the microbiome complex, interaction and communication of the microorganisms is a prerequisite. One example showing the importance of interkingdom communication is the interaction of *Candida albicans* and bacteria being essential to prevent microbial pathogenesis (Manos 2022), although the molecular basis of the interactions and communication is not fully understood. Furthermore, nutrient competition and metabolic interaction also affect the strength of a complex microorganism community. The growth and survival of a wide variety of microorganisms—for example, in the gut—are influenced by highly fluctuating environmental conditions (Newton et al. 2026). The use of medications such as antibiotics or the consumption of certain foods leads to perturbations and a shift in the composition of microorganisms in the gut. These changes in the gut microbiome are also accompanied by competition for nutrients and alteration in cross-feeding structures (Culp and Goodman 2023; Newton et al. 2026). Thus, further research is necessary to understand interaction and communication in microbial consortia better and to use the acquired knowledge for controlled application of microbial communities.

The use of controlled communication of consortia of microorganisms will widen the range of possible applications in synthetic biology and biotechnological processes and be helpful for expanding a green and sustainable chemical production (Liu et al. 2020). To distribute the burden of production in a consortium of microorganisms will overcome the limitations of single-strain processes. Furthermore, the potential of microbial consortia in sustainable agriculture, bioremediation and plant diseases has been pointed out recently (Nunes et al. 2024).

Bacterial populations mainly communicate via quorum sensing mechanisms. The signal molecules are autoinducers 1 or 2, acyl-homoserine lactones or autoinducing peptides respectively (Boo et al. 2021). Furthermore, diffusible signaling factors (DSF) are known as signaling molecules mediating interkingdom communication among microbes, fungi, plants, insects and zebrafish (He et al. 2023; Wu et al. 2025). DSF belong to a newly characterized family named diffusible signaling factor family (DSFF), which are unsaturated fatty acids and are widespread in Gram-negative bacteria (He et al. 2023; Wijesinghe et al. 2025).

The communication via signaling molecules is a prerequisite for bacterial communities to adjust behavior, e.g., for biofilm formation, bioluminescence, and environmental challenges (Liu et al. 2025). An example for controlled artificial interspecies communication between the Gram-negative bacterium *Escherichia coli* and the Gram-positive *Bacillus megaterium* using quorum sensing peptides was successfully established by Marchand and Collins (Marchand and Collins 2013). The authors used autoinducing peptides coded by the AgrD gene of *Staphylococcus aureus* and the respective AIP-secretion system heterologously expressed in *E. coli*. The respective receptor and signal transduction system was expressed in *B. megaterium*, where the green fluorescent protein was set under the control of a quorum sensing peptide inducible promoter. The successful interspecies communication was demonstrated.

**Fig. 1:**
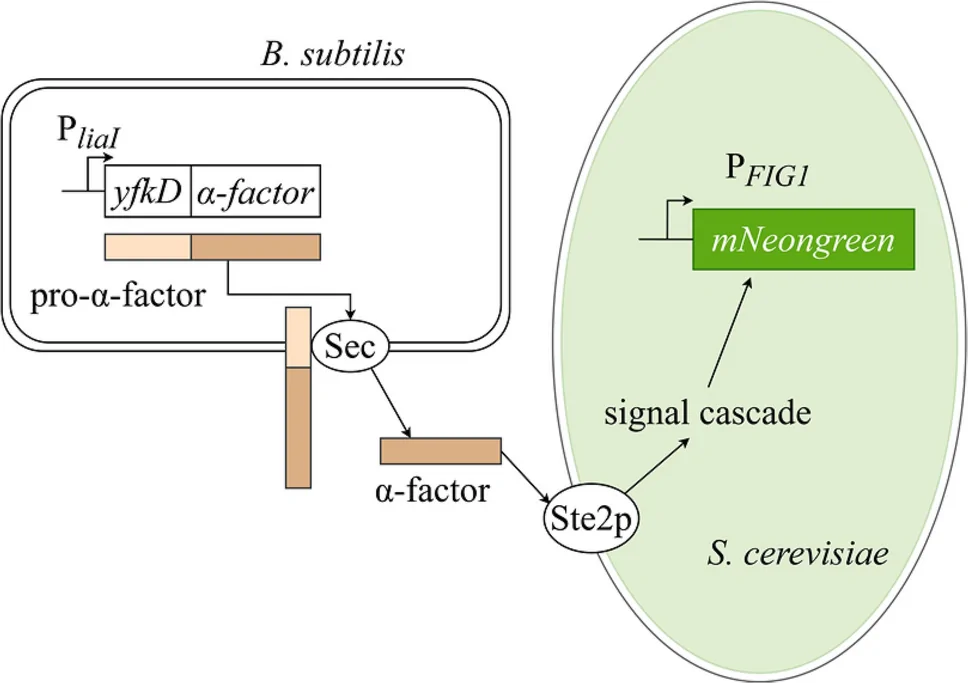
Schematic presentation of signal transduction between *B. subtilis* and *S. cerevisiae*. *B. subtilis* was engineered to express and secrete α-factor through native Sec secretion system using the secretion signal peptide of protein YfkD. The secreted α-factor is recognized by the Ste2p receptor membrane protein on the surface of *S. cerevisiae* and initiates the pheromone signal cascade. The signal cascade activates P_*FIG1*_ and thereby the expression of mNeongreen fluorescence

Moreover, we recently demonstrated the successful controlled interkingdom communication between the yeast *Saccharomyces cerevisiae* and *Bacillus subtilis* (Vološen et al. 2024). *S. cerevisiae* was engineered to heterologously produce the quorum sensing peptides CSF and PhrF of *B. subtilis*. The activity of the peptides was measured in *B. subtilis* reporter strains in which bioluminescence or fluorescence was activated in the presence of the peptides. In contrast to bacteria yeasts communicate via small pheromone peptides. Haploid *S. cerevisiae* cells exist as a cell of mating type α or a and divide by mitotic division (Herskowitz 1988). They secrete small peptides as pheromones: a- and α-factor, respectively. The peptides bind to specific receptors on the surface of cells of the opposite mating type, activating a signal cascade which leads to morphological changes, cell cycle arrest and the expression of specific genes (Leberer et al. 1997). Among these genes the *FIG1* gene is induced about 100-fold already 20 min after receptor activation (Erdman et al. 1998). We exploited this system already to generate an intraspecies communication and signal amplification system in *S. cerevisiae* (Groß et al. 2011). We either used pheromones for establishing interspecies communication between *S. cerevisiae* and *Schizosaccharomyces pombe*, which exhibits an analogous mating type system (Hennig et al. 2015). The above-mentioned interkingdom communication via quorum sensing peptides was directed from yeast to bacteria. To establish the communication from *B. subtilis* to *S. cerevisiae*, cells of *B. subtilis* heterologously expressing and secreting the yeast pheromone α-factor were generated by using a recently developed signal peptide toolbox for *B. subtilis* (Vološen et al. 2025). As signal receiver, yeast cells were constructed harboring the coding sequence for the fluorescence protein mNeongreen under control of the *FIG1* promoter. Here we show that *B. subtilis* upon induction is able to express and secrete a functional α-factor leading to the expression of mNeongreen in yeast receiver cells. This is the first example, to our knowledge, of a bioengineered controlled interkingdom communication between the Gram-positive bacterium *B. subtilis* and the eukaryotic yeast *S. cerevisiae.*

## Material and methods

### Strain growth conditions

The strains used in this study are listed in Table 1. Briefly, *S. cerevisiae* was cultivated in liquid yeast peptone dextrose (YPD) medium (10 g/L yeast extract, 20 g/L peptone, and 20 g/L glucose; pH 6.5) at 30 °C under continuous shaking. *B. subtilis* strains were grown in LB medium (5 g/L yeast extract, 10 g/L peptone, and 10 g/L sodium chloride) under aeration at 37 °C. Cultures on solid medium were treated the same way without agitation. For *B. subtilis* TMB6428, kanamycin (10 µg/mL) and chloramphenicol (5 µg/mL) were added for selection.

**Table 1:** Strains used in this study

Strain name	Genotype	Source
*S. cerevisiae* BY4741 *∆bar1 ∆far1::FIG1_mNeongreen*	*MATa his3D1 leu2D0 met15D0 ura3D0 YIL015w::kanMX4 YJL157c::FIG1_mNeongreen_TEF_HIS3*	*This study*
*B. subtilis* W168	Δ*trpC2*, wild type	Lab strain
*B. subtilis* WB800N	*nprE aprE epr bpr mpr*::*ble nprB*::*bsr* Δ*vpr wprA*::h*yg* (Neo^R^)	Mobitec, Göttingen
*B. subtilis* TMB6428	WB800N *amyE*::pBS1C-P_*liaI*_-*yfkD*-α-pheromone_H_	Vološen et al. 2025

### Co-cultivation of *B. subtilis* and *S. cerevisiae*

Co-cultures of *S. cerevisiae* and *B. subtilis* were grown in LB-medium supplemented with 20 g/L glucose at 31.5 °C with shaking. Co-cultures were set up with a ratio of 1:6, meaning OD_600_ of *S. cerevisiae* was set to 0.1 and OD_600_ of *B. subtilis* to 0.6 as starting OD. Co-cultures were incubated for 3 h prior to induction with 100 µg/mL bacitracin (Sigma Aldrich, Taufkirchen, Germany) or 250 nM synthetic α-factor (Zymo Research, Freiburg, Germany). As a negative control, bacitracin was added to the yeast-only culture, too. Samples were taken directly upon induction and after 2, 4, and 22 h of cultivation. All experiments were carried out in triplicates in Erlenmeyer flasks filled to a maximum of 10% of the total volume.

### *S. cerevisiae* strain construction

Genomic integration of the construct *FIG1_mNeongreen* into the genome of *S. cerevisiae* BY4741 *∆bar1 ∆far1* was done by homologous recombination. For that, primers with overlapping regions to the *far1*-Locus were designed (see Table 2). As a selective marker, the *HIS3* gene, coding for imidazole glycerol-phosphate dehydratase (His3p), was used under the control of the constitutive *TEF*-promoter. The desired construct (overlap far1_FIG1_mNeongreen_TEF_His3_overlap far1) was generated by using overlap-extension PCR with subcloning into the pJet1.2/blunt vector. *FIG1*_*mNeongreen_cyc-term* was synthesized by ATG Biosynthetics (Merzhausen, Germany) and amplified by using primers 1 and 3 (see Table 2). *TEF*_*his3_tef_term* was amplified from vector pFA6-His3MX6 (Longtine et al. 1998) using primers 4 and 2. The final construct was amplified by using primers 1 and 2. About 1 µg of the PCR-product was used for yeast transformation. Yeast transformation was done according to Gietz and Woods (Gietz and Woods 2006). In brief, *S. cerevisiae* BY4741 *∆bar1 ∆far1* was cultivated until it reached an OD_600_ of ~ 2. Then, 2 mL of the culture was harvested (3000 × g, 5 min, 4 °C) and supernatant was discarded. The pellet was washed with 1 mL of ddH_2_O and 1 mL of 100 mM lithium acetate by centrifugation as mentioned above. The pellet was layered with 240 µL 50% (w/v) PEG3350, 36 µL 1 M lithium acetate, 25 µL single-stranded DNA (herring sperm-DNA, 10 mg/mL) and 1 µg of PCR-product in a final volume of 50 µL. Next, the reaction mixture was vortexed for 1 min and afterwards incubated for 30 min at 30 °C with shaking, following a heat shock at 42 °C for 20 min. The transformed cells were centrifuged as mentioned above, the pellet was resuspended in 100 µL of sterile distilled water and plated on W0 selection medium (1.9 g/L yeast nitrogen base; 5 g/L ammonium sulfate ((NH_4_)_2_SO_4_); 20 g/L glucose; 1.5 g/L agarose) lacking the amino acid histidine. Cells were incubated for 2–3 days at 30 °C. Several clones were pooled and plated on a new selection medium plate. Genomic DNA was isolated and the position of the gene of interest (GOI) was verified by PCR. After verification of the right genome position, the strain was named *S. cerevisiae* BY4741 *∆bar1 ∆far1::FIG1_mNeongreen* and cultivated in YPD medium with no need for further selection.

**Table 2:** List of primers used for *S. cerevisiae* strain construction

#	Primer name	Sequence 5′−3^*a*^
1	Far1_Fig1_ for	ACTGGAAAGCTTCGTGGGCGTAAGAAGGCAATCTATTATTGAATGATCAACCAAACGCCGATATAG
2	Tef_term_Far1_rev	CGTATGTGAATGCTGGTCGCTATACTGTAGTTCGGGAATCGAGGCCCGTATTTCGAGGCT
3	Cyc_term_OE_rev	GGGACGCTCGAAGGCTTTAATTTGCTAATTAAGGCGCGCCAGATCTG
4	TEF_prom_OE_for	CGCTCGAAGGCTTTAATTTGCTAATTAAGGCGCGCCAGATCTGTTTAG

^*a*^Underlined sequences correspond to the *FAR1* overlapping regions

### Western Blot analysis

For Western Blot analysis, 2 mL of co-culture samples were taken at the depicted time points and centrifuged at 3,500 × g for 5 min, 4 °C. Supernatant was discarded and pellets resuspended in varying amounts of phosphate-buffered saline (PBS, pH 7.4) depending on pellet size. Isolation of total protein was done by adding glass beads (⌀ 0.25–0.5 mm) to the sample pellets and mixing them in a beat mill for 8 min at 30 Hz. Total protein concentration was determined by using the BioRad *DC* Protein Assay-Kit (Feldkirchen, Germany). For every sample, 20 µg of total protein was loaded on a denaturing SDS-glycine polyacrylamide gel (5% stacking gel, 12% separating gel). After the gel run, proteins were blotted on a 0.45 µm PVDF membrane or stained with colloidal Coomassie staining solution. Detection of protein of interest (POI) was done by using anti-mNeongreen (1:5,000; Agrisera, Vännäs, Sweden) as primary antibody and anti-rabbit-HRP (1:5,000; GE Healthcare UK) as secondary antibody.

### Flow cytometry

For flow cytometry, 1 mL of the co-culture samples was taken and directly measured. For that, samples were vortexed rigorously, and 5–200 µL (depending on sample density) were added to approx. 2.5 mL of Sheath fluid and mixed. Measurement was done with a PARTEC (now Sysmex) CyFlow SL device with the following settings: measurement of 20,000 cells after flow rate was stable between 150 and 300 cells/s; forward scatter (FSC) gain: 100; side scatter (SSC) gain: 140; fluorescence channel (FL1) gain: 500. Mean fluorescence value was calculated from 20,000 cells; no gating was performed to separate yeast and bacteria.

### Microscopy

Epifluorescence microscopy was performed using a Keyence BZ-X810 inverted microscope (Keyence, Frankfurt/Main, Germany). 1 mL samples were taken at the depicted time points and centrifuged for 8 min at 3,500 × g. Supernatant was discarded, and the pellet was resuspended in 500–1000 µL of PBS. Meanwhile, 1% (w/v) agarose in water was tempered to 54 °C in a water bath. 15 µL of warm agarose was pipetted on an objective slide and covered with a cover glass until it solidified. The cover glass was removed, and 10 µL of the co-culture samples was added to the agar block and covered with a fresh cover glass. Fluorescence microscopy was then performed with the GFP filter (excitation 472.5/30 nm, emission 520/35 nm) using a 40 × objective. Exposure time for fluorescence detection was 2 s.

### Statistical analysis

Statistical evaluation for flow cytometry data was done using a two-sided Student’s *t*-test for the comparison of mean values of uninduced and induced samples. Significance values were as follows: *p* ≥ 0.05 (n.s.; not significant); *p* < 0.05 (*; significant); *p* < 0.01 (**; highly significant); and *p* < 0.001 (***; very high significant).

## Results

### Pheromone expression and detection in single strains

To enable interkingdom communication the chosen strains of *B. subtilis* and *S. cerevisiae* had to be genetically modified, aiming *B. subtilis* to produce and secrete yeast mating pheromone α-factor and *S. cerevisiae* to react to α-factor by expressing the green fluorescent protein mNeongreen. *B. subtilis* pheromone-producing strain was developed as previously described in (Vološen et al. 2025). An evaluation vector, established in Vološen et al. 2025, was used to first introduce the codon-optimized α-factor gene sequence. In the next step, the signal peptide of the gene *yfkD*, responsible for α-factor secretion, was introduced in front of α-pheromone. Lastly, the bacitracin-inducible promoter P_*liaI*_ was introduced to drive the expression of pro-α-factor (Fig. 1). The developed plasmid was integrated via homologous recombination into the *amyE* locus of the protease-deficient strain *B. subtilis* WB800N, which finally resulted in the pheromone producer strain TMB6428 (see Table 1). To test the developed secretion system, a culture of *B. subtilis* TMB6428 was induced with 100 µg/mL bacitracin. Secreted peptide concentration was analyzed via ELISA (also see Figure S1). Although the concentration of the secreted pheromone was lower than industry standards (up to 8 nM), its production in *B. subtilis* TMB6428 was consistent and robust (Vološen et al. 2025). Thus, we used TMB6428 as α-pheromone producer in co-cultivation with the α-pheromone sensor yeast strain to check and investigate cell–cell communication. To construct the α-pheromone sensor strain, we used the haploid yeast strain *S. cerevisiae* BY4741 *Δbar1 Δfar1*, which contains deletions in the *BAR1* and *FAR1* loci. *BAR1* codes for an aspartyl protease that degrades and inactivates the α-factor. *FAR1* encodes for a cyclin-dependent kinase inhibitor. Deletion leads to yeasts lacking the G1-growth arrest after pheromone induction. Especially, the *BAR1* deletion is helpful for the successful artificial induction of the yeast cells using α-factor. Genomic integration of mNeongreen under the control of α-inducible *FIG1*-promoter was done by homologous recombination of the construct into the *FAR1* locus (Fig. 1). Genomic integration of mNeongreen in yeast was necessary as *B. subtilis* is not able to grow properly in yeast minimal medium, which would have been needed to select for the episomal mNeongreen-carrying plasmid via auxotrophy in yeast cells. The response of the constructed yeast strain to α-factor induction was first tested with artificial α-factor. mNeongreen fluorescence was observable already after 2 h of incubation at 30 °C under shaking conditions (see Figure S2).

### Co-cultivation of engineered *B. subtilis* and *S. cerevisiae*

To co-cultivate *B. subtilis* producer and *S. cerevisiae* sensor cells*,* a cultivation medium fulfilling the needs of both organisms had to be identified. After testing different media, the most appropriate was a combination of standard LB medium with the addition of 2% (w/v) glucose, yeast’s main carbon source. Pre-cultures were set up in LB medium for *B. subtilis* and YPD medium for *S. cerevisiae*. Next, a cell density ratio between the two organisms had to be defined to overcome potential growth problems in co-cultivation. *B. subtilis* cells have a doubling time of approximately 30 min, whereas haploid *S. cerevisiae* cells grow slower with a doubling time of approx. 90 min in nutrient-rich medium (Burdett et al. 1986; Sherman 2002). Another issue was that the authentic gene *MFα* in yeast coding for intrinsic yeast α-factor contains 4 copies of the peptide. After translation, the pro-peptide is processed to 4 single α-factor peptides, which are secreted. The engineered *B. subtilis* producer strain will produce only one copy of the α-factor; hence the expected heterologous secreted α-concentration will be low (Vološen et al. 2025). Therefore, a higher cell density of *B. subtilis* (= higher number of cells) could be advantageous to adjust the amount of produced α-factor in co-culture. Additionally, we expected a low mNeongreen fluorescence signal compared to a high-copy plasmid-based yeast strain, as there is only one copy of the *mNeongreen* gene integrated in the genome. Previous experiments with eGFP as a reporter fluorescence cassette failed due to too low fluorescence intensity, which is why we decided to use the mNeongreen fluorescent protein as reporter to enhance the fidelity of the fluorescence signal. Due to these facts, we set the starting OD_600_ of *S. cerevisiae* to 0.1 and OD_600_ of *B. subtilis* to 0.6, maintaining a ratio of approx. 1:6. This ratio was proven to be successful.

### Proof of principle: induction of α-pheromone sensitive *S. cerevisiae* by pheromone secreting *B. subtilis*

**Fig. 2:**
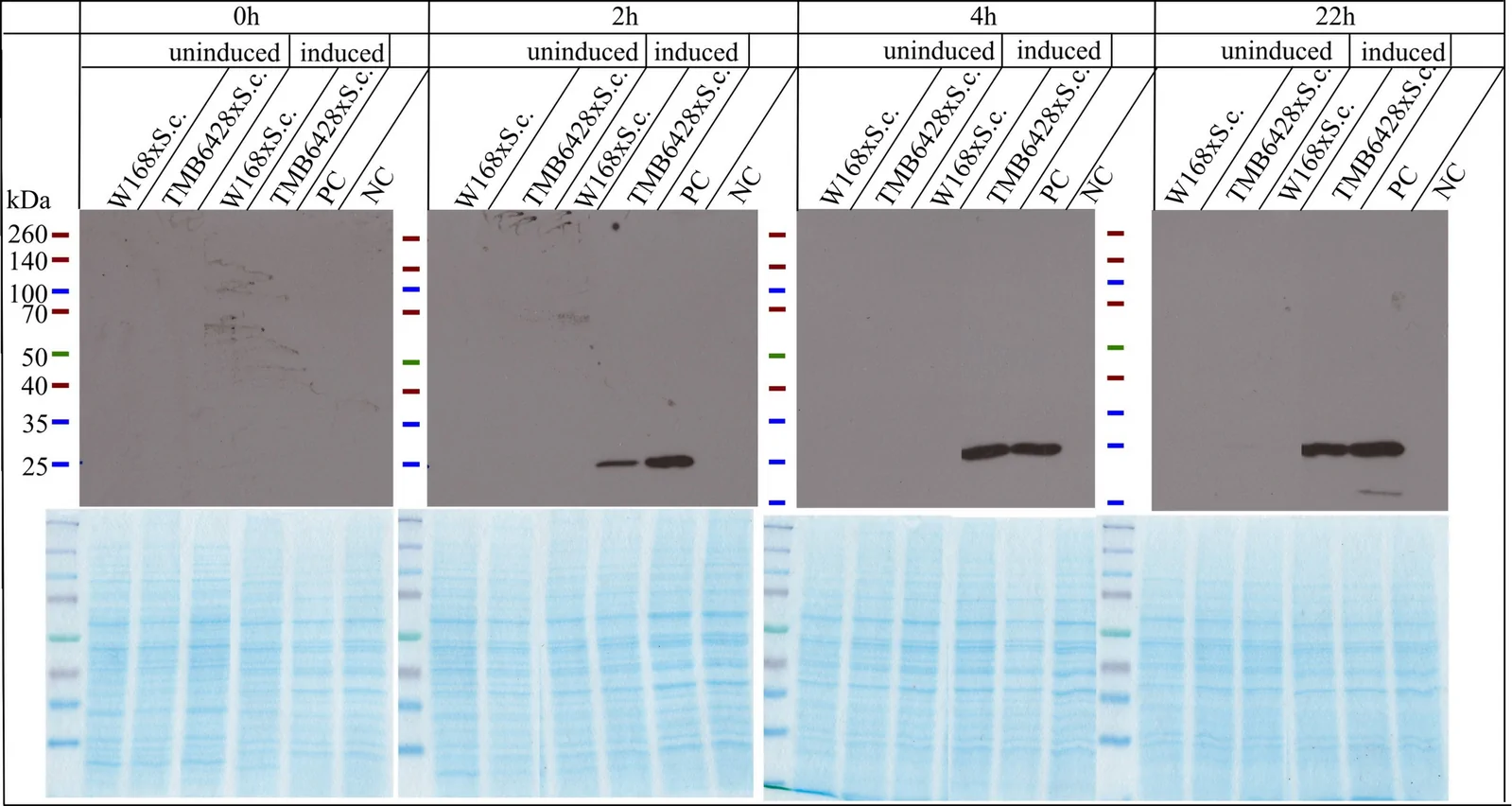
Western Blot analysis of *B. subtilis* and *S. cerevisiae* co-cultures. Western Blot (upper panel) was done by using mNeongreen-antibody. Lower panel shows Coomassie-stained SDS-PAGE gels. Induction of co-cultures was done in mid-exponential phase with 100 µg/mL bacitracin. Abbreviations: W168xS.c.: co-culture of *B. subtilis* W168 (wild type) with *S. cerevisiae* BY4741 *∆bar1 ∆far1::FIG1_mNeongreen*; TMB6428xS.c.: co-culture of *B. subtilis TMB6428* (α-factor producer) and *S. cerevisiae* BY4741 *∆bar1 ∆far1::FIG1_mNeongreen*; PC: positive control, *S. cerevisiae* BY4741 *∆bar1 ∆far1::FIG1_mNeongreen* induced with 250 nM synthetic α-factor; NC: negative control, *S. cerevisiae* BY4741 *∆bar1 ∆far1::FIG1_mNeongreen* without induction; kDa: kilodalton; expected size of mNeongreen = 26.6 kDa

To establish co-culture signaling and interkingdom communication, we cultivated bacteria and yeast until the mid-exponential growth phase. Then bacitracin was added to induce α-factor expression in *B. subtilis* TMB6428. Samples were taken at certain time points and analyzed for the existence of mNeongreen by Western Blot analysis and flow cytometry. Western Blot analysis was performed to verify the expression of mNeongreen and thus indirectly prove the successful signal transduction between *B. subtilis* and *S. cerevisiae* (Fig. 2).

Western Blot results showed successful expression of mNeongreen in co-culture samples with α-factor producing *B. subtilis* as well as in samples containing yeast only and induced with synthetic α-factor (Fig. 2; 2, 4 and 22h). Negative controls, meaning uninduced co-cultures and yeasts, as well as co-cultures with *B. subtilis* W168 (not able to produce α-factor), showed no detectable mNeongreen signal. Furthermore, mNeongreen proteins were already detected two hours after induction with bacitracin. Here, signal intensity in the co-culture is a bit lower than in the positive control, leading to the conclusion that the starting concentration of *B. subtilis* produced α-factor is lower than the concentration of synthetic α-factor used for positive control (PC). After 4 h, signal intensity of co-culture sample increased, proving an efficient activation of the yeast pheromone signaling cascade by *B. subtilis* produced α-factor. Therefore, the presence of mNeongreen protein indirectly proves successful α-factor production in *B. subtilis*. Western Blot analysis does not give any information about the functionality of the produced mNeongreen protein. Therefore, we performed flow cytometry and microscopy to prove the expression of a native protein by measuring its fluorescence.

**Fig. 3:**
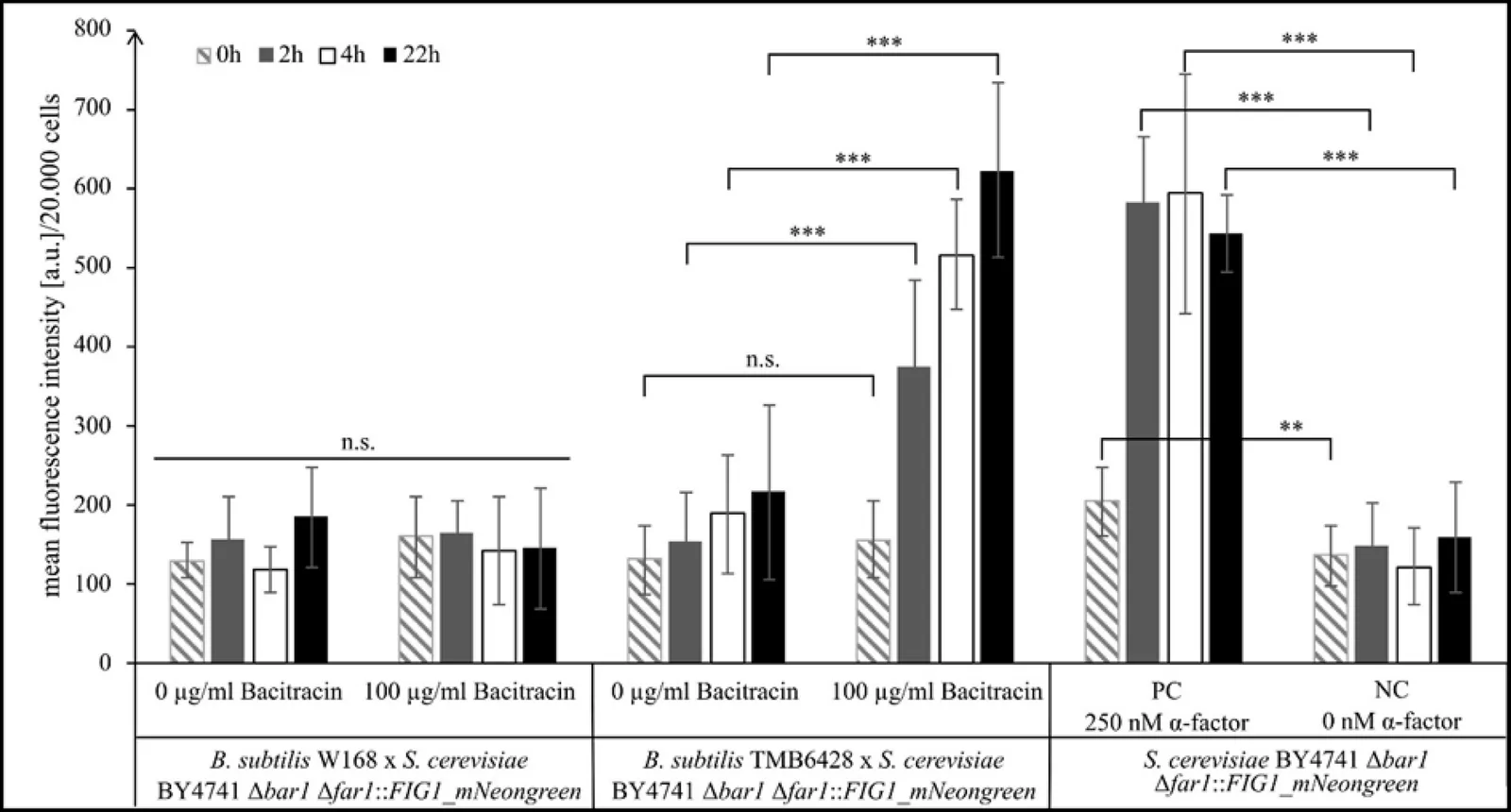
Flow cytometry of *B. subtilis* and *S. cerevisiae* co-cultures. Mean fluorescence intensity values of 20,000 cells measured in biological triplicates (6 biological replicates for co-cultures with TMB6428) are shown. For each biological replicate, technical triplicates were measured. Statistical evaluation for flow cytometry data was done using a two-sided Student’s t-test for the comparison of mean values of uninduced and induced samples. Significance values were as follows: *p* ≥ 0.05 (n.s.; not significant); *p* < 0.05 (*; significant); *p* < 0.01 (**; highly significant); *p* < 0.001 (***; very high significant). PC = positive control; NC = negative control

With flow cytometry, we measured the fluorescence signal of 20,000 cells in total and calculated the mean value. No gating for fluorescence channel was performed as the gain for the forward scatter was chosen in a way to exclude the *B. subtilis* signal from the measurement. Results of flow cytometry measurement showed successfully an engineered interkingdom control between *B. subtilis* and *S. cerevisiae* (Fig. 3). While cells of the wild-type *B. subtilis* W168 were not able to trigger expression of mNeongreen in the yeast cells, the α-factor producing *B. subtilis* strain TMB6428 did after induction with bacitracin. Two hours after induction, a significant mNeongreen fluorescence is measured compared to the uninduced strain. Over time, the signal is getting stronger, indicating an increasing concentration of α-factor in the co-culture. In the uninduced co-culture sample with α-factor producing *B. subtilis* TMB6428, there is also a slight increase in signal intensity visible after 4 and 22 h (Fig. 3). This is reasoned by an already known slight leakiness and intrinsic transition state induction of P_*liaI*_ (Jordan et al. 2007). In comparison to that, the yeast-only positive control culture induced with synthetic α-factor does not show the time-dependent increase in mNeongreen fluorescence signal. Here, α-factor is not provided over time but only once at the beginning of the experiment; therefore, the α-concentration cannot increase, which could explain this result. All uninduced negative controls show comparable mean fluorescence values. Standard deviations between biological replicates are quite high. This could be explained by the natural variation in organism fitness, depending on the age of the plate cultures used and also the medium quality, which could vary from batch to batch.

**Fig. 4:**
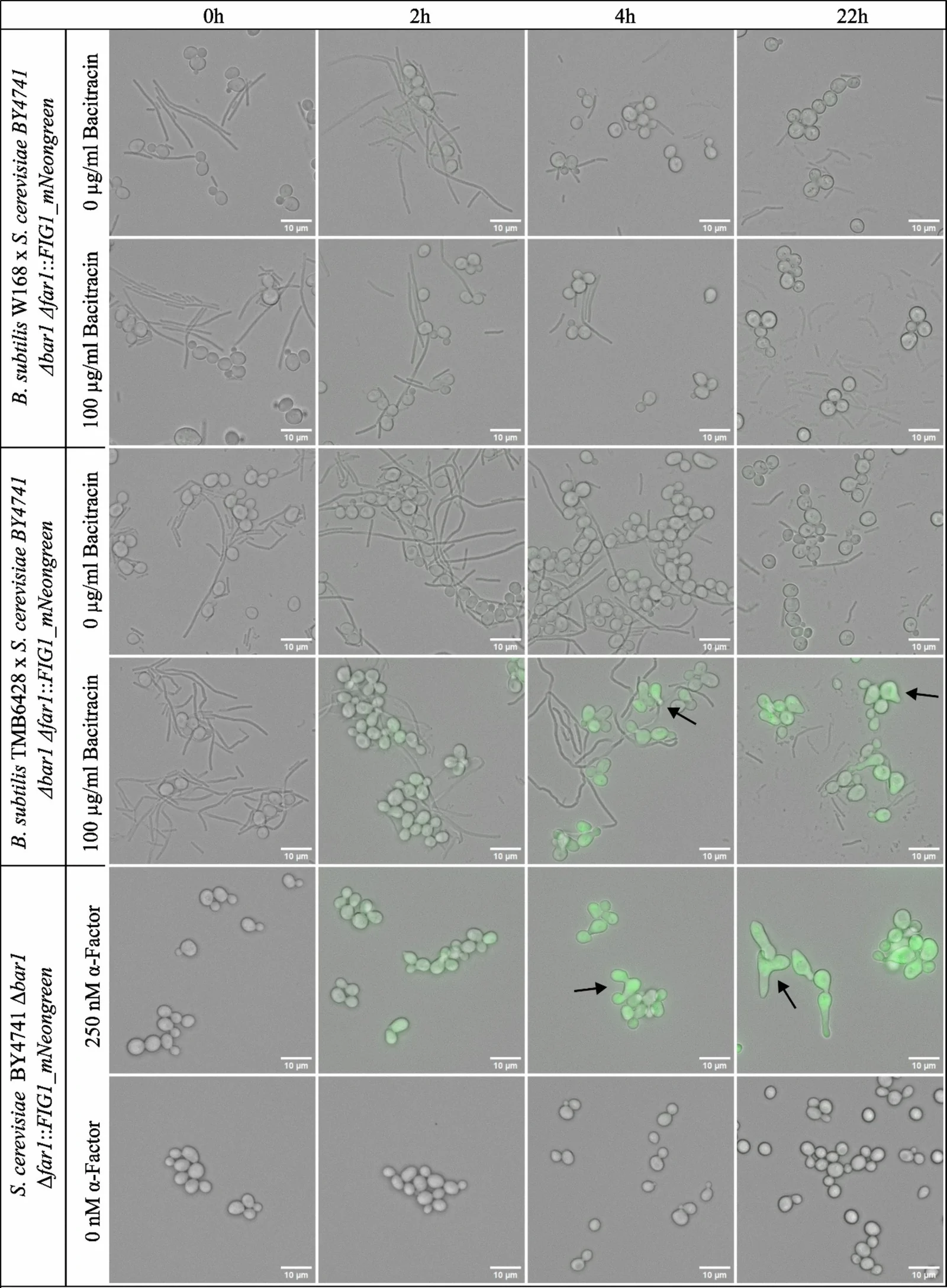
Fluorescence microscopy of *B. subtilis* and *S. cerevisiae* co-cultures. Overlay images of brightfield and fluorescence channel images with an exposure time of 2 s for fluorescence images at the depicted time points are shown. Arrows show examples for shmoo formation. Scale bar: 10 µm

Taken together, flow cytometry data already assume the achievement of establishing an artificial interkingdom signaling. However, visualization of co-culture signaling was performed with epifluorescence microscopy to investigate the brightness of the mNeongreen signal and estimate the co-culture’s healthiness. Additionally, microscopy allows us to confirm the presence of *S. cerevisiae* cells in a “shmoo”-formation (Cross 1988). “Shmoo” is the morphological change of growth existing in the presence of α-pheromone (Fig. 4, see arrows). In uninduced samples of co- and single cultures, cells are normal in size and round shaped. Additionally, under induced condition morphology of *B. subtilis* cells is not changing as well. In general, α-factor induced yeast cells start to show “shmoo”-formation 4 h after induction, which increases over time within 22 h after induction. Fluorescence signal of mNeongreen is detectable already two hours after adding bacitracin or synthetic α-factor to the cultures, supporting the findings by flow cytometry and Western Blot analysis. “Shmoo”-formation and mNeongreen fluorescence point out a successful induction of the pheromone-dependent pathway in *S. cerevisiae*, which is a result of the heterologous expression and secretion of functional α-factor in *B. subtilis*. Uninduced co-culture of TMB6428 with yeast also shows a faint green fluorescence 4 and 22 h after induction, which is due to promoter leakiness, as already mentioned. In contrast, no fluorescence of yeast cells is observable when in co-culture with *B. subtilis* wild type.

In summary, all results presented here confirm that a controlled bioengineered interkingdom communication between *B. subtilis* and *S. cerevisiae* is successfully established and might be a good starting point for further applications.

## Discussion

The results of this study nicely enlarge our recent findings within an establishment of interkingdom signaling between *Saccharomyces cerevisiae* and *Bacillus subtilis* (Vološen et al. 2024). In line with our published cell–cell communication from yeast to *Bacillus subtilis *via the small quorum sensing peptides CSF and PhrF, we successfully showed here that artificial heterologous expression and secretion of the eukaryotic peptide α-pheromone in bacteria works and results in cellular responses of *Saccharomyces cerevisiae*. On the one hand, we demonstrate the activation of the α-pheromone induced P_*FIG1*_ promoter in yeast sensor cells by artificial secreted α-pheromone from *B. subtilis*. It has been already known that yeast´s P_*FIG1*_ responses very fast and up to 100-fold increased activity to α-pheromone (Erdman et al. 1998; Groß et al. 2011). Here, we used flow cytometry and Western Blot to detect and quantify the reporter fluorophore mNeongreen as indicator for *FIG1* promoter activation by α-factor. Originally, mNeongreen was found in the lancelet *Branchiostoma lanceolatum* and is characterized as the brightest green fluorescent protein that is known so far (Deheyn et al. 2007). mNeongreen shows a higher stability to photo bleaching than GFP and its derivatives during fluorescence microscopy procedures. Furthermore, the maturation time of mNeongreen is shorter compared to GFP, which makes mNeongreen more suitable for reporter assays (Hostettler et al. 2017; Shaner et al. 2013). On the other hand, we also wanted to prove if the heterologous expressed and secreted α-pheromone of *B. subtilis* is working efficiently in terms of the induction of mating physiology in *S. cerevisiae*. Fluorescence microscopy of co-cultivated yeast and bacteria induced with bacitracin not only showed the already observed fluorescent mNeongreen but also uncovered the presence of “shmoo”-formed yeast cells. This cell morphology of haploid cells of *S. cerevisiae* is typical during α- and a-factor mating processes (Tkacz and MacKay 1979). Here, the formation of “shmoo” cells indicated the successful heterologous secretion of fully functional α-pheromone from *B. subtilis* that is physiological active in yeast cells. Moreover, *B. subtilis* was able to secrete heterologous expressed α-pheromone in sufficient concentrations to induce mating differentiation processes in *S. cerevisiae*. Producer strain TMB6428, which was used in this study, is able to provide up to 8 nM α-pheromone in one hour after induction with bacitracin (Vološen et al. 2025). After 2 h in co-cultivation of the bacitracin-induced producer strain together with the sensor yeast strain, we observed first signals of mNeongreen by using flow cytometry, microscopy and Western Blot. As positive control, we always used 250 nM synthetic α-factor together with the sensor yeast cells and observed brighter signals of mNeongreen after two hours compared to the signals from co-cultivation. We expected such results in terms of a higher expression and brightness of mNeongreen because the use of 250 nM α-pheromone is very excessive. Under normal physiological conditions, e.g., cultivation of *S. cerevisiae* yeast cells depleted for *BAR1*, addition of 100 nM synthetic α-factor was sufficient to induce mating (Groß et al. 2011). Another positive result of this study was the successful cell–cell signaling in co-cultivation of *B. subtilis* and *S. cerevisiae* in liquid media where the cells come in contact to each other. We already co-cultivated both microorganisms for the first time in liquid MV-media—which is very complex in composition—in our recent study (Vološen et al. 2024). Here, we used the most common and easy to handle LB medium supplemented with glucose to improve the growth of *S. cerevisiae*. This led us to conclude that direct mixed co-cultivation of yeast and *B. subtilis* is comparably easy and cheap when cultivation is characterized by an optimized cell ratio of both microorganisms, incubation temperature is set at ± 30 °C and a commonly used medium like LB supplemented with glucose is used.

In the future, we aim to establish a functional synthetic “sensor-actuator” co-cultivation circuit in which engineered *B. subtilis* cells serve as sensor for certain signal substances (e.g., antibiotics) that induce the production of yeast’s pheromones. These, by *B. subtilis*, secreted small signaling pheromones in turn will activate the expression of proteins or enzymes (e.g., to degrade antibiotic substances) in yeast cells. The ability of *S. cerevisiae* to produce antibiotic degradation enzymes in response to artificially added α-pheromone has been described recently (Pohl et al. 2025). Furthermore, the accumulation of degradation end products can be used as negative feedback loop in a self-contained circuit to shut down the peptide/protein expression in sensor and actuator cells intrinsically and without external intervention.

## Supplementary Information

Below is the link to the electronic supplementary material.

ESM 1 (DOCX 127 KB)

## Data Availability

No datasets were generated or analysed during the current study.
