# Supplementary material for: Interkingdom communication 2.0: pheromone-based control between Bacillus subtilis and Saccharomyces cerevisiae
Source: Appl Microbiol Biotechnol. 2026 Oct 15;110:310. doi: 10.1007/s00253-026-14069-5 (PMC13672771; doi:10.1007/s00253-026-14069-5)
Supplement: Supplementary file 1 [file 253_2026_14069_MOESM1_ESM.docx]

**Supplemental Information**

**Figure S1** Measurement of α-pheromone concentration by using indirect ELISA. Concentration was determined 0 and 1 hour after induction of the strain TMB6428 with 100 µg/mL bacitracin. To quantify α-factor concentration, a protocol for indirect ELISA was adopted from Hennig et al. (Hennig et al., 2016). In brief, 100 µL of culture supernatant was transferred to each well of the ELISA plate. Additionally, 100 µL of synthetically produced α-factor (Peptides & Elephants GmbH (Berlin, Germany) was added in a concentration range from 0.21 nM to 50 nM to develop a four-parameter dose-response calibration curve. Next, the plate was covered with a lid and stored at room temperature for 2 h. After 2 h, wells were washed three times with 200 µL of PBST buffer and residual binding sites on ELISA plates were blocked with 100 µL of 1 % (w/v) BSA (bovine serum albumin) dissolved in PBST buffer for 2 h at room temperature. ELISA plate was rewashed three times with PBST buffer, and 100 µL of 0.4 µg mL-1 α-factor antibody from rabbit (Peninsula Laboratories International, Inc. (currently BMA Biomedicals, Switzerland)) dissolved in 0.5 % BSA in PBST buffer was added. After 1h, wells were rewashed, and 100 µL of Anti-Rabbit IgG (H+L) HRP Conjugate (working solution 1:2500 diluted in 0.5 % BSA in PBST buffer) (Promega) was added. After 1h, the last washing step was performed and peroxidase reaction started by adding 100 µL of Pierce™ TMB substrate kit solution in each well. The reaction was stopped by adding 100 µL of 2 M sulfuric acid. Subsequently, absorbance was measured at 450 nM in a Synergy Neo2 Hybrid Multimode Microplate Reader from BioTek (Winooski, VT, USA). The experiment was performed as technical triplicate. Error bars represent the standard deviation from the mean value.

**Figure S2** Fluorescence of mNeongreen in synthetically induced and uninduced *S. cerevisiae* BY4741 *∆bar1 ∆far1::FIG1_mNeongreen* cells measured by flow cytometry*.* Mean fluorescence values of 20,000 cells at the indicated timepoints (0h, 2h, 4h, 6h, 8h, 24h) are shown. Error bars show standard deviations of the fluorescence signal intensity of 20,000 cells. Cultures were grown either induced with 500 nM synthetic α-factor (+α) or uninduced without synthetic α-factor (-α)
